# Leaf fitness and stress response after the application of contaminated soil dust particulate matter

**DOI:** 10.1038/s41598-022-13931-6

**Published:** 2022-06-16

**Authors:** Marie Lhotská, Veronika Zemanová, Milan Pavlík, Daniela Pavlíková, František Hnilička, Marek Popov

**Affiliations:** 1 Department of Botany and Plant Physiology, Faculty of Agrobiology, Food and Natural Resources, Czech University of Life Sciences Prague, Kamýcká 129, 165 00 Praha 6, Czech Republic; 2 Department of Agro-Environmental Chemistry and Plant Nutrition, Faculty of Agrobiology, Food and Natural Resources, Czech University of Life Sciences Prague, Kamýcká 129, 165 00 Praha 6, Czech Republic

**Keywords:** Physiology, Plant sciences

## Abstract

In this study, we observed the effect of the application of soil dust enriched with risk elements (Cd, Pb, As and Zn) to leaf surfaces of lettuce (*Lactuca sativa* var. *capitata*) while it was grown under hydroponic conditions. This study aimed to determine how low soil dust particulate matter (PM) doses affected the activity of or damaged the photosynthetic apparatus and how the uptake of risk elements was associated with both epigenetic changes (5-methylcytosine content, i.e., 5mC) and stress metabolism. During the study, we obtained many results pertaining to risk element contents and biochemical (total phenolic content (TPC), malondialdehyde (MDA) content and the amount of free amino acids (AAs)) and physiological (photosynthesis parameters: net photosynthetic rate, transpiration rate, intercellular CO_2_ concentration, stomatal conductance, instantaneous water-use efficiency, maximum quantum yield of PSII, chlorophyll and carotenoid contents, and leaf water potential (WP)) plant features. The results showed an increase in MDA and 5mC. However, the transpiration rate, WP and free AAs decreased. In conclusion, contamination by very low doses of soil dust PM had no direct or significant effect on plant fitness, as shown by the TPC and 5mC content, which indicates that plants can overcome the oxidative stress caused by the accumulation of risk elements. From the above, we propose the use of epigenetic changes as biomarkers of potential changes in the activation of plant metabolism under stress caused by environmental pollution.

## Introduction

The sources of risk elements in dust can be from natural localities as well as from localities with anthropogenic contamination due to current or past mining activities. The problems caused by dust applied to plant leaves have been examined in many studies^1,2^. Risk elements can be absorbed by leaves *via* wet or dry deposition of atmospheric fallout^3^. The absorption and accumulation of risk elements can influence crop safety and human health^4^.

As published by Salih and Aziz^5^, leaves are considered good bioindicators of air pollution. According to Bendel and Weigel^6^, short-term exposure to soil dust particulate matter (PM) at relatively high concentrations generally leads to acute visible foliar injury. Chronic exposure to lower concentrations results in alterations in physiological and biochemical processes that may manifest as chlorosis, premature senescence, growth and yield reductions. The absorption and effects of PM are influenced by foliar morphology and physiology, e.g., cuticular waxes, stomatal numbers, frequency and length of trichomes and length of petioles, as well as physicochemical properties of PM, such as the particle size, the content of toxic active ingredients, solubility, volatility, polarity, reactivity, hydrolysis, the stability to ozone, UV radiation and atmospheric oxygen^7–9^. The level of damage that can be caused by dust depends on plant resistance against dust particles and on the ecotoxicological availability of the dust through exposure processes^7^. Of course, the whole effect is dependent on the resistance, tolerance or sensitivity of the plant^9^.

Dust has different physicochemical properties that determine bioavailability and thus toxicokinetic processes^4,10^. Dust particles can change the optical properties of a leaf, especially the surface reflectance in the visible and shortwave infrared radiation range^11^. Soil dust features are especially influenced by their origin^12^. Therefore, soil dust can contain metals/metalloids such as Cd, Pb, Zn and As that can accumulate in plant leaves after foliar application^13^. These risk elements in dust can pose a threat to plants by causing oxidative stress through an imbalance in reactive oxygen species (ROS) production^14,15^. However, Turan et al.^16^ showed a positive role of Zn in minimising the negative effects of ROS because Zn is the main cofactor of the antioxidative enzyme superoxide dismutase^17^. The uptake of contaminants affects the primary metabolism of plants^18,19^. A study by Gajbhiye et al.^2^ showed that the results and consequences of foliar uptake must be derived from the interactive role between plant and metal type. In addition, the dose of contaminant plays an important role. Despite considerable progress in recent years about the foliar absorption of risk elements by plants, there still exists a gap in knowledge^3^. Soil dust particles can enter the inside of a leaf by a stomatal or cuticular pathway^13^. If the PMs are so large that they do not pass through the stomata, they can clog them^20^. The persistence of particles is also dependent on weather conditions. Rainwater or wind may remove these particles^21^. Wet surfaces increase deposition potential, notwithstanding that rain may partly carry away the deposited dust^22^.

The dust particles on leaf surfaces limit stomatal function^23^, increase temperature^24^, contribute to some physiological changes^25^ and cause changes in photosynthetic pigment ratios^26^ and mineral contents^27^. Atmospheric compounds and air pollutants that interact with other abiotic and biotic factors have a complex effect on crop performance^6^ as well as soil quality^28^.

The main aim of our experiment was to determine the influence of low soil dust application on lettuce leaves growing under defined conditions. The experimental plants were grown under hydroponic conditions to eliminate the effect of soil^29^ and the absorption of risk elements by roots. The effects of risk elements in soil dust PMs, which cause stress after absorption by leaves, were investigated in the lettuce plants. Therefore, we determined how the uptake of risk elements was associated with both epigenetic changes and stress metabolism and how low soil dust doses affected the activity/damage of the photosynthetic apparatus.

## Materials and methods

### Plant material and hydroponic experiment

A hydroponic pot experiment was carried out under greenhouse conditions (natural photoperiod; temperature day/night 23/19 °C; relative humidity ~ 60%; light and dark period 10/14 h) using lettuce (*Lactuca sativa* var. *capitata*). After pre-cultivation in Rockwool, lettuce plants were grown in 15 l standard Hoagland solution for 14 days. Two treatments were designed: control and soil dust application. On the leaves of the treatment with soil dust, 1 g of sieved soil (mesh size 1 × 1 mm) from a contaminated location (Table 1) was applied by dusting, and then rain was simulated by the spraying of demineralized water (10 ml/plant). In the experiment, there were twelve plants per pot per treatment. The plants were harvested in four replicates per treatment (three plants were randomly selected per replicate). After harvesting, the leaves were washed with demineralized water, dried by cellulose wadding and partitioned, with one portion being immediately frozen in liquid nitrogen and kept in a deep freezer (− 80 °C) until DNA isolation and free AA analysis, while the second portion was oven-dried to a constant weight (three days at 40 °C) and homogenised for elemental analysis.

**Table 1:** Characteristics of the contaminated soil used for soil dust application.

Location	Litavka (49° 43′ N, 14° 0′ E)
Soil type and subtype	Gleyic fluvisol
Soil texture	Clay loamy sand
pH (–)	5.3
CEC (mmol_(+)_/kg)	57 ± 4
C_org_ (%)	1.8
As_total_ (mg/kg)/As_ws_ (mg/kg)	286 ± 14/1 ± 0.2
Cd_total_ (mg/kg)/Cd_ws_ (mg/kg)	37 ± 0.8/0.2 ± 0.02
Pb_total_ (mg/kg)/Pb_ws_ (mg/kg)	2344 ± 57/5 ± 1
Zn_total_ (mg/kg)/Zn_ws_ (mg/kg)	3515 ± 54/16 ± 1

CEC is the cation exchange capacity, C_org_ is organic carbon, (As, Cd, Pb or Zn)_total_ is the pseudototal content of elements determined by ICP–MS (extraction by aqua regia), and (As, Cd, Pb or Zn)_ws_ is the water-soluble content of elements determined by ICP–OES (24-h extraction by demineralized water, 1:5 w/v). Czech legislation limits for pseudototal contents of elements in light-textured soils (mg/kg): As—15, Cd—0.4, Pb—55 and Zn—105^30^.

### Analysis of elements

Homogenised plant material (0.5 ± 0.05 g of dry weight) was digested in 10 ml of a 4:1 (v/v) mixture of HNO_3_ and H_2_O_2_ in an Ethos 1 device (MLS GmbH, Germany). After cooling, the digested sample was diluted to 50 ml with demineralized water. The contents of As, Cd, Mg, Pb and Zn were determined using an inductively coupled plasma-optical emission spectrometer (ICP–OES; Agilent 720, Agilent Technologies Inc., USA). A certified reference material (CRM NIST 1573a tomato leaves) was mineralized under the same conditions for quality assurance.

### Determination of lipid peroxidation

The malondialdehyde (MDA) content was measured based on a modified thiobarbituric acid (TBA) method^31^. Fresh leaves (0.4 g) were homogenised with liquid nitrogen and 80% ethanol and centrifuged in 2 ml microcentrifuge tubes for 5 min at 6000 rpm. Aliquots of 0.7 ml of each supernatant were mixed with 0.7 ml of 0.65% TBA in 20% TCA (trichloroacetic acid) and 0.01% BHT (butylated hydroxytoluene), and a second set of 0.7 ml samples was mixed with 0.7 ml 20% TCA and 0.01% BHT. The microcentrifuge tubes were incubated at 95 °C for 25 min, and after cooling, they were centrifuged for 5 min at 6000 rpm. The absorbance at 440 nm, 532 nm, and 600 nm was read on a UV–Vis spectrophotometer (Evolution 210, Thermo Scientific), and the concentration of MDA (nmol/g FW) was calculated using the equation of Heath and Packer^32^.

### Determination of the total phenolic content

The total phenolic content (TPC) was determined according to the original method described by Singleton and Rossi^33^, with slight modifications, using ethanolic extracts from the lipid peroxidation assay with tenfold-diluted Folin–Ciocalteu reagent (Sigma–Aldrich) and a UV–Vis spectrophotometer (Evolution 210, Thermo Fisher Scientific) at wavelength 760 nm. The content was calculated as equivalents of gallic acid (GAE mg/g FW).

### Isolation of DNA and determination of relative DNA methylation status based on the % of 5-methylcytosine

Plant DNA was isolated by a NucleoSpin Plant II Molecular Kit (Macherey–Nagel GmbH & Co. KG, Germany) as described by Zemanová et al.^34^. The global DNA methylation status of DNA was determined using 100 ng of isolated DNA and a MethylFlash Methylated DNA Quantification Kit (Fluorometric; Epigentek Group Inc., Farmingdale, USA) according to the manufacturer’s instructions. A spectrophotometer (Tecan Infinity M200, Tecan Deutschland GmbH) with excitation at 530 nm was used to measure the fluorescence at 590 nm using the Magellan program.

### Analysis of free amino acids

Free amino acids (AAs) were extracted, derivatized and quantitated as described by Pavlíková et al.^35^. Extracts were derivatized using an EZ:faast kit (Phenomenex, USA). The prepared samples were analysed on a Hewlett Packard 6890 N/5975 MSD gas chromatography–mass spectrometry (GC–MS) system (Agilent Technologies, USA).

### Determination of gas-exchange parameters

The portable gas exchange system LCpro+ (ADC BioScientific, Ltd., UK) was used for in situ determination of the net photosynthetic rate (P_n_; µmol/CO_2_/m^2^/s), transpiration rate (E; mmol H_2_O/m^2^/s), intercellular CO_2_ concentration (Ci; µmol CO_2_/mol), stomatal conductance (g_s_; mol H_2_O/m^2^/s) and calculation of instantaneous water-use efficiency (WUE; WUE = P_n_/E). All measurements were conducted between 8:00 and 11:30 Central European Time (CET). The duration of each individual measurement was 10 min after the establishment of steady-state conditions inside the measurement chamber. The conditions in the chamber were 25 °C, ambient CO_2_ concentration (550 ± 50 µl/l), airflow rate of 205 ± 30 µmol/s and photosynthetically active radiation of 650 ± 50/µmol/m^2^/s.

### Determination of pigments

The pigment content in the leaves was measured by a UV–Vis spectrophotometer (Evolution 210, Thermo Fisher Scientific Inc., USA). Fresh leaf segments (0.5 cm^2^) were incubated in the dark in 1 ml dimethylformamide, with shaking for 24 h. The absorbance of the extract was measured at wavelengths of 480, 646.8 and 663.8 nm. Absorbance values at 710 nm were subtracted from these measurements. From these data, pigment contents were calculated using the Porra et al.^36^ equations for chlorophylls and the Wellburn^37^ equations for carotenoids:

$$\begin{aligned} & {\text{Chlorophyll}}\;a\;\left( {{\text{Chl}}\;a;\;\mu {\text{g/ml}}} \right){:}\;{\text{Chl}}\;a = {12}.0 \times {\text{A663}}.{8}{-}{3}.{11} \times {\text{A646}}.{8} \\ & {\text{Chlorophyll}}\;b{:}\;\left( {{\text{Chl}}\;b;\;\mu {\text{g/ml}}} \right){:}\;{\text{Chl}}\;b = {2}0.{78} \times {\text{A646}}.{8}{-}{4}.{88} \times {\text{A663}}.{8} \\ & {\text{Total}}\;{\text{chlorophyll}}\;\left( {{\text{Chl}}_{{\text{t}}} ;\;\mu {\text{g/ml}}} \right){:}\;{\text{Chl}}\;a + {\text{Chl}}\;b = {17}.{67} \times {\text{A646}}.{8} + {7}.{12} \times {\text{A663}}.{8} \\ & {\text{Carotenoids}}\;\left( {{\text{Car}};\;\mu {\text{g/ml}}} \right){:}\;{\text{Car}} = \left( {{1}000 \times {\text{A48}}0{-}{1}.{12}\;{\text{Chl}}\;a{-}{34}.0{7}\;{\text{Chl}}\;b} \right)/{245} \\ \end{aligned}$$

The pigment contents were normalised to the leaf area.

### Determination of fluorescence

Chlorophyll fluorescence was measured using a portable fluorometer (OS1-FL; Opti-Sciences, ADC, BioScientific, Ltd., UK). A fresh leaf was shaded for 20 min using clips to set up a dark-adapted state. Chlorophyll fluorescence was excited by a 660 nm solid-state light source, with filters blocking radiation longer than 690 nm. The saturation of the measured photosystem was achieved by using a filtered 35 W halogen lamp (350–690 nm) with a pulse of 15,000 µmol/m^2^/s for 0.8 s. The maximum quantum yield of PSII (F_v_/F_m_) was calculated as F_v_/F_m_ = (F_m_ − F_0_)/F_m_.

### Determination of the leaf water potential

Leaf water potential (WP; MPa), as a measure of the energy status of the water in a system, was measured using a dew point PotentiaMeter (Decagon Devices, Inc., Pullman, USA). The leaves of the plants were placed in a disposable syringe, the air was drawn off from the syringe, and the syringe was tightly closed with Parafilm. The specimen was frozen at –18 °C, and then thawed, and the sap was pushed into the measuring chamber of the PotentiaMeter.

### Statistical analyses

Statistical analyses were performed with Statistica 12.0 software (StatSoft, USA). All data were checked for homogeneity of variance and normality (Levene’s and Shapiro–Wilk tests). One-way analysis of variance (ANOVA) followed by a post hoc comparison with Fisher’s LSD test (*p* ≤ 0.05) was used to identify significant differences between treatments. Correlation analysis was performed using Pearson’s linear correlation (*r*; *p* ≤ 0.05). The present study complies with relevant institutional, national, and international guidelines and legislation.

## Results

### Content of risk elements in leaf biomass and their influence on leaf yield

The application of soil dust influenced the dry weight (DW) of the leaves as shown in Table 2. The plant DW in the soil dust PM treatment was 16.7% lower than that of the control, but the difference was not significant. The contents of the analysed risk elements were under the limit of detection in the control except for Zn (Table 2). The application of soil dust increased the contents of As, Cd, Pb and Zn in the dust treatments 126-, 780- and 309.5-fold, respectively, while Zn was only threefold higher compared with the control. The highest increase from these risk elements was shown for Cd. All mentioned risk elements correlated with each other (*r* = 0.95–0.99; *p* ≤ 0.05). The application of soil dust increased the content of the analysed risk elements in the leaves, but the leaf yield was not significantly decreased by the toxicity of these elements.

**Table 2:** Contents of risk elements and leaf yields in the control and soil dust treatments.

Parameter	Control	Soil dust
As (mg/kg DW)	< 0.03	3.79 ± 0.65
Cd (mg/kg DW)	< 0.001	0.78 ± 0.21
Pb (mg/kg DW)	< 0.02	6.19 ± 1.37
Zn (mg/kg DW)	36.49 ± 7.62^a^	111.22 ± 15.76^b^
DW (g)	10.80 ± 2.23^a^	9.03 ± 0.06^a^

DW represents the dry weight per treatment. Values are means ± SD (*n* = 4). Lower-case letters indicate significant differences based on Fisher’s LSD test (*p* ≤ 0.05). Data with the same letter are not significantly different.

### Leaf stress response after the application of contaminated soil dust particulate matter

**Figure 1:**
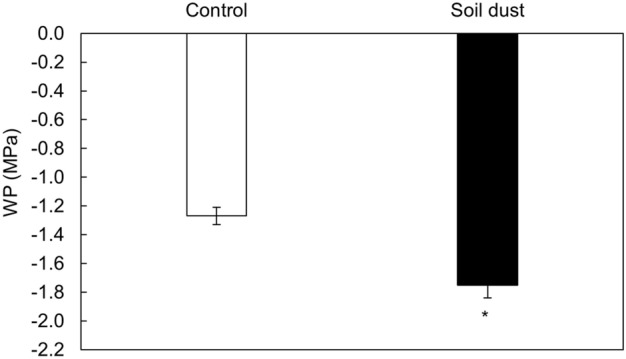
The effect of soil dust application on leaf water potential (WP) in lettuce grown under hydroponic conditions. Significant differences between treatments are indicated by asterisks (ANOVA; Fisher’s LSD test; *p* ≤ 0.05).

The application of soil dust to leaf area decreased leaf WP compared with the control (Fig. 1). The average values of leaf WP in the dust and control treatments were − 1.75 MPa and − 1.27 MPa, respectively. In comparison with the control, the soil dust treatment leaf water potential was decreased by 27.4%.

**Figure 2:**
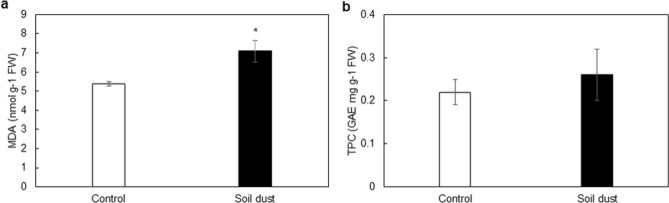
The effect of soil dust application on the content of malondialdehyde (MDA) (**a**) and the total phenolic content (TPC) (**b**) in lettuce grown under hydroponic conditions. Significant differences between treatments are indicated by asterisks (ANOVA; Fisher’s LSD test; *p* ≤ 0.05). Columns without asterisks are not significantly different.

MDA is a well-known bioindicator of the degree of membrane damage because it is caused by oxidative stress after the degradation of unsaturated fatty acids. The application of soil dust increased the content of MDA in the dust treatment by 24.1% compared to the control (Fig. 2a); however, the increase in TPC caused by the application of soil dust PM to leaves in the dust treatment was not significant compared to the control (Fig. 2b).

**Figure 3:**
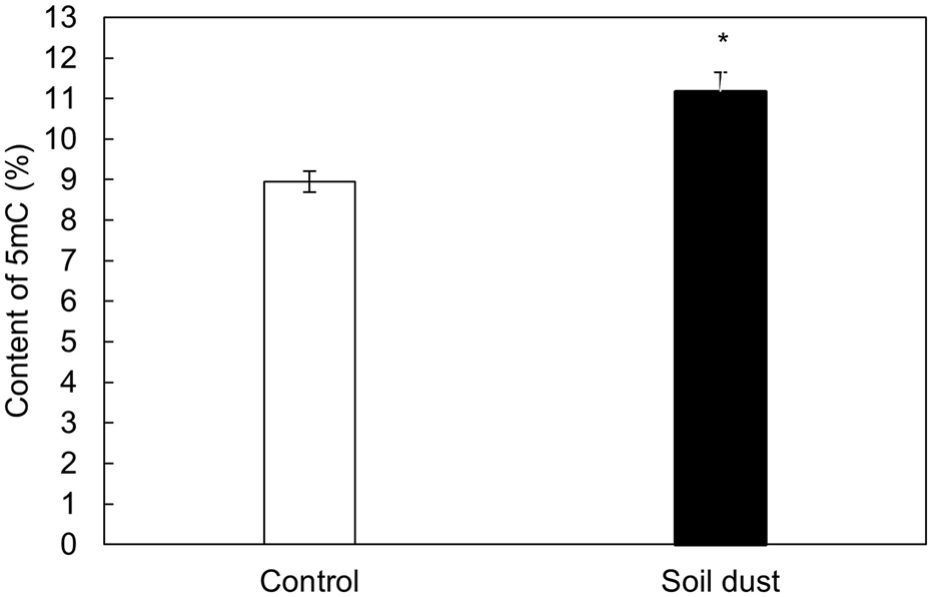
Effect of the soil dust application on the content of 5-methylcytosine (5mC) in lettuce grown under hydroponic conditions. Significant differences between treatments are indicated by asterisks (ANOVA; Fisher’s LSD test; *p* ≤ 0.05).

Monitoring epigenetic changes in plants is an innovative way to characterise the response of plants to the effects of abiotic and biotic stress factors. The content of 5mC influenced by soil dust application to leaves is shown in Fig. 3. The content was significantly higher in the dust treatment than in the control. The 5mC content increased by 24.9%. It is clear from the increasing methylation of DNA that the toxicity of the absorbed risk elements was not relevant based on the insignificant reduction of the DW leaf yield.

The free AA content was affected by soil dust application, i.e., there were changes in the free AA metabolism of the lettuce. Soil dust application decreased the total content of free AAs, which consisted of 19 free AAs and related compounds, by 20.4% compared with the control (Table 3). Furthermore, the content of transport and storage AAs (tAAs), which contain glutamate (Glu), glutamine (Gln), aspartate (Asp) and asparagine (Asn), was lower in the soil dust treatment than in the control; however, the difference was not significant (Table 3). The results clearly showed an effect of soil dust application on free Glu, free proline (Pro) and free ornithine (Orn) in lettuce leaves when compared with the control, and the contents of these AAs decreased by 15%, 12% and 11.5%, respectively. The content of tAAs correlated with the total free AA content in the control (*r* = 0.97, *p* ≤ 0.001) and in the soil dust application treatment (*r* = 0.90, *p* ≤ 0.01). A higher correlation was observed between the total free AA content and Pro (*r* = 0.99, *p* ≤ 0.001) in the soil dust application treatment than in the control (*r* = 0.89, *p* ≤ 0.01). The same trend was observed for the total free AA content and Orn (*r* = 0.99, *p* ≤ 0.001 and *r* = 0.91, *p* ≤ 0.01 under soil dust application and in the control, respectively) (Table 3).

**Table 3:** Content of selected free amino acids in the lettuce leaves of the control and soil dust treatments.

Parameter	Control	Soil dust
Σ AAs (µmol/kg FW)	10,380.61 ± 1645.33^b^	8259.34 ± 850.99^a^
tAAs (µmol/kg FW)	2752.09 ± 925.89^a^	2095.11 ± 177.44^a^
Glu (µmol/kg FW)	857.01 ± 127.88^b^	735.08 ± 69.82^a^
Pro (µmol/kg FW)	439.73 ± 42.37^b^	387.17 ± 43.80^a^
Orn (µmol/kg FW)	575.30 ± 55.09^b^	508.93 ± 59.38^a^

Σ AAs: total content of free amino acids, tAAs: sum of transport and storage free amino acids, Glu: free glutamine, Pro: free proline, Orn: free ornithine. Values are means ± SD. Lower-case letters indicate significant differences based on Fisher’s LSD test (*p* ≤ 0.05). Data with the same letter are not significantly different.

### Response of the photosynthetic apparatus after application of contaminated soil dust particulate matter

The application of soil dust to the leaf area affected some gas-exchange parameters (Table 4). Soil dust decreased the E of plants, which was 22.2% lower in the dust treatment than in the control. In contrast, the application of soil dust increased the WUE by 31.6% compared with the control. Furthermore, other gas-exchange parameters were affected by soil dust application, but the results of P_n_, Ci and g_s_ were not significant (Table 4). The application of soil dust to the leaves did not affect the fluorescence parameter (Table 4) because the decrease in the maximum quantum yield of PSII in the dust treatment was not significant compared to the control. These results show that the effect of toxic elements in dust on photosynthetic parameters is consistent with an insignificant reduction in the DW of the leaves.

**Table 4:** Gas-exchange parameters and chlorophyll fluorescence in the control and soil dust treatments.

Parameter	Control	Soil dust
P_n_ (µmol CO_2_/m^2^/s)	9.34 ± 2.05^a^	9.75 ± 0.95^a^
E (mmol H_2_O/m^2^/s)	1.79 ± 0.44^b^	1.42 ± 0.21^a^
g_s_ (mol H_2_O/m^2^/s)	0.16 ± 0.05^a^	0.16 ± 0.03^a^
Ci (µmol CO_2_/mol)	340.55 ± 22.71^a^	335.28 ± 13.90^a^
WUE (µmol CO_2_/mmol H_2_O)	5.24 ± 0.74^a^	6.91 ± 0.81^b^
F_v_/F_m_ (–)	0.83 ± 0.01^a^	0.83 ± 0.01^a^

P_n_: net photosynthetic rate, E: transpiration rate, g_s_: stomatal conductance, Ci: intercellular CO_2_ concentration, WUE: water use efficiency (P_n_/E), F_v_/F_m_: maximum quantum yield of photosystem II (F_v_/F_m_ = (F_m-_F_0_)/F_m_). Values are means ± SD. Lower-case letters indicate significant differences based on Fisher’s LSD test (*p* ≤ 0.05). Data with the same letter are not significantly different.

Of the pigments extracted by dimethylformamide, only the contents of Chl *a* and Chl_t_ were significantly lower in the dust treatment than in the control (Table 5). The contents were decreased by 26.4% and 23.5%, respectively. Chl *a*/*b* and Chl_t_/Car were calculated from the measured values. The difference between the Chl *a/b* ratio in the soil dust treatment and the control was 13.5% (3.62 and 3.13, respectively). The value of the Chl_t_/Car ratio in the soil dust treatment decreased by only 4.6% compared with the control (4.8 and 4.6, respectively). Neither ratio differed significantly. Furthermore, the content of Mg was significantly decreased by 9.1% compared with the control (Table 5).

**Table 5:** Contents of chlorophyll, carotenoids and magnesium in the control and soil dust treatments.

Parameter	Control	Soil dust
Chl *a* (mg/m^2^)	22.65 ± 2.49^b^	16.69 ± 1.10^a^
Chl *b* (mg/m^2^)	6.27 ± 0.85^a^	5.44 ± 1.08^a^
Chl_t_ (mg/m^2^)	28.92 ± 3.31^b^	22.13 ± 2.09^a^
Car (mg/m^2^)	6.07 ± 0.96^a^	4.88 ± 0.79^a^
Mg (g/kg DW)	4.37 ± 0.32^b^	4.00 ± 0.24^a^

Chl *a*: chlorophyll *a*, Chl *b*: chlorophyll *b,* Chl_t_: total chlorophyll, Car: carotenoids. Values are means ± SD. Lower-case letters indicate significant differences based on Fisher’s LSD test (*p* ≤ 0.05). Data with the same letter are not significantly different.

## Discussion

According to Maletsika and Nanos^38^, the application of dust originating from contaminated soil increases the aboveground biomass. In contrast, the low dose of applied soil dust in the present study did not affect the DW yield of the leaves in the two treatments (Table 2), while the risk elements contained in soil dust applied to the leaf surfaces increased the content of elements in the plant biomass (Table 2), similar to the results reported by Xiong et al.^8^. Additionally, the study of Gajbhiye et al.^2^ showed a link between the application of soil dust to the foliar surface, plant growth and the content of elements in the leaves. The results of Žalud et al.^39^ showed that the application of a PM suspension to leaf surfaces increased the content of the elements, including Cd. According to these authors, the effect of PM size on the foliar absorption of elements was negligible.

Heavy metals such as Cd^40,41^ and Pb^19^ and metalloids such as As^41^ can cause oxidative stress because they induce an increase in reactive oxygen species (ROS) production at the subcellular level and increase MDA production^14^. The plant responses described above are in perfect agreement with our results (Fig. 2). Oxidative stress and associated stress metabolism are secondarily induced by soil dust PM^42^. Our results showed that lettuce leaves respond to risk elements in soil dust PM after the absorption of these elements into the leaf (Fig. 2, Table 2). Increased MDA is a product of oxidative stress, which subsequently increasing leaf senescence, including premature tissue ageing^43^, by signalling molecules^44^.

Zn, Cu, Mn, Fe and Ni play key roles as cofactors of various metalloenzymes^45^. During oxidative stress, these elements, especially Zn together with Mn, Cu and Fe, play a role in the mitochondrial, chloroplast and cytosolic enzyme superoxide dismutase, which is irreplaceable in the plant antioxidant defence system^17^. Zinc also plays an important role in the synthesis of photosynthetic pigments^46^. In this experiment, a high amount of As and very high amounts of Zn and Pb were measured in the applied soil dust (Table 1). A high Zn content in the analysed plants compared to the control (Table 2) was therefore available for the functioning of the antioxidant system, which is an integral part of the ascorbate–glutathione cycle^45^. Therefore, the presence of Zn has a positive effect by reducing the oxidative stress caused by Cd^47^. Thus, a significant decrease in Chl *b* and Car was not confirmed (Table 5).

The content of photosynthetic leaf pigments is reduced by the presence of some risk elements in dust PM^5^. A negative effect on the content of pigments, especially chlorophyll, occurred in soybean due to Cd^14^, in *Pistia stratiotes* due to Pb^48^ and in sunflower due to As^49^. However, the opposite result was reported by a very important study conducted by Gusman et al.^50^, who examined the effect of As on lettuce pigments. The increased content of risk elements defines the response of a plant’s fitness costs, such as the pigment contents, photosynthetic parameters and epigenetic changes^34^. Therefore, these plant responses are determined by the adaptation/resistance/tolerance/sensitivity of the plants to different contents of toxic elements. When a plant depletes its C resources, pigments are reduced, and negative changes in photosynthetic parameters are observed^51^. Another important indicator of plant fitness cost is the accumulation of bioactive forms of cytokinins that delay senescence and increase chlorophyll biosynthesis^52^. Nevertheless, the *Pteris* hyperaccumulator continues to accumulate both As and bioactive and transport forms of cytokinins, which delay senescence by activating chlorophyll biosynthesis. However, even if the C sources are depleted and chlorophyll fluorescence decreases, irreversible leaf senescence is induced^35^. It follows from the above that the presented results of the photosynthetic parameters (Table 4) and contents of photosynthetic pigments (Table 5) indicate reversible leaf senescence^53^.

The decline in the chlorophyll content also has a close connection with the decrease in Mg^27^ because there was a significant difference in the Mg content of the lettuce leaves between the soil dust treatment and the control (Table 5). This is consistent with the published results of Ciećko et al.^54^, who showed a negative correlation between Cd and Mg. Magnesium deficiency and concomitant accumulation of risk elements in lettuce, compared to the control, reduced both the chlorophyll content and the photosynthesis parameters, as discussed earlier in two *Noaccaea* species^55^. This is also in agreement with the results of Küpper et al.^18^, who showed that in plants, Mg^2+^ is substituted in chlorophyll by heavy metals (Cd, Cu, Ni, Zn and Pb). This replacement, i.e., Cd, leads to the inhibition of photosynthesis and, in particular, to the breakdown of chlorophyll. At the same time, Cd after penetration into chloroplasts is known to disrupt their function and inhibit enzymes associated with chlorophyll biosynthesis and photosystem II responses^40^.

ROS accumulation degrades unsaturated fatty acids^56^ into a number of products such as oxylipins, carbonyls and reactive electrophilic products. These include odd-carbon metabolites such as MDA, 2-propenal, propanal, 2-pentenal and others, which are potential precursors of odd fatty acids^57^ or 12-oxo-phytodienoic acid and jasmonic acid^58^. At the same time, high levels of peroxidation of unsaturated bonds reduces the content of unsaturated fatty acids, the decrease of which causes a violation of the permeability and fluidity of the biomembrane^59^.

Increased ROS production in chloroplasts causes a reduced rate of photosynthesis processes (e.g., CO_2_ fixation), damage to thylakoid function and ultrastructural changes in the thylakoid system in barley or tomato chloroplasts by Pb^19^ and in lettuce by Cd^60^. Because of the action of accumulated ROS, plant growth is inhibited, photosynthetic pigments are reduced, the photosynthetic apparatus is destroyed and nutrient homeostasis is impaired.

Subsequently, stress phytohormones (e.g., jasmonic, salicylic and abscisic acids) and antioxidant secondary metabolites are induced^61^. One group of important antioxidant secondary metabolites is phenolic compounds (Fig. 2b), which include phenylpropanoids, lignans, naphthoquinones, stilbenes, flavonoids, isoflavones and others^62^. In lettuce, phenolic compounds (Fig. 2b) and other parameters that did not show significant differences in the reduction of P_n_, g_s_, Ci, Chl *b*, Car, F_v_/F_m_, and DW (Tables 2, 4 and 5) conflict with the increase in MDA (Fig. 2a) as well as damage to the photosynthetic apparatus (E, WUE, Chl *a*; Tables 4 and 5). This indicates only partial damage to the photosynthetic apparatus and partial activation of the antioxidant plant system^63^, which explains the insignificant reduction in antioxidant phenolic metabolites (Fig. 2b). This is also consistent with the results of Agati et al.^64^, who linked an increase in phenolic compounds to a decrease in growth by a reduction of the growth phytohormone auxin.

Increases/decreases in phenolic compounds are related to the duration of stress and the degree of damage of the photosynthetic apparatus, induction of the onset of reversible/irreversible leaf senescence and potential changes in epigenetic gene silencing that cause gene inactivation/reactivation after DNA methylation/demethylation^65^. The results showed that there was no significant reduction in most of the photosynthetic parameters and pigments. The effect of stress caused by toxic elements after the application of soil dust PM on lettuce leaves was small, and therefore, the lettuce plants had a good fitness status. Lettuce fitness and increased DNA methylation (Fig. 3) are associated with efforts to increase growth, while DNA demethylation is associated with decreased growth, accelerated flowering and leaf senescence^66^. Changes in DNA methylation in seedlings are very specific because as these very young leaves age, DNA methylation must increase, which in turn leads to increased growth of the examined seedlings^67^. The increase in 5mC also supports the fact that the application of very low doses of Cd to seedlings first increases DNA methylation, while after a further increase in Cd there is already a decrease in DNA methylation^68^. In plants, an increase/decrease in % 5mC and good/poor fitness, as well as the degree of stress caused by the accumulated toxic elements, are inextricably linked^34,35^.

Due to the application of soil dust, WUE increased even though the transpiration rate was limited by narrowed stomata. This probably affected the decrease in the amount of CO_2_ in mesophyll cells and subsequently decreased the photosynthetic performance. This is in line with the results of the foliar application of Zn on wheat, which enhanced WUE^69^. However, the other observed risk elements caused the opposite effect. For example, Chaneva et al.^70^ stated that the transpiration rate rapidly decreased in the presence of Cd in hydroponic systems. Similar results were found in soybean and lettuce in the presence of Pb^71^, which is assumed to be connected to root damage. The next element with the same impact is As. Arsenic limits stomatal conductance, thus decreasing the transpiration rate^50^. Maletsika and Nanos^38^ carried out a similar study on plants exposed to dust. From their results, it is obvious that the applied dust had a negative influence on the transpiration and photosynthetic rates as well as stomatal conductance and the chlorophyll content. From the above results, it is clear that the composition of the risk elements in the applied soil dust (Table 1) influenced synergistic and antagonistic relationships between the risk elements, thus affecting the resulting increase/decrease in WUE.

Although dust did not have a direct effect on leaf water potential^72^ in the present study, a significant decrease in leaf WP was observed in the soil dust treatment (Fig. 1). The explanation is the same as that for transpiration and WUE. The decrease in leaf WP could be explained by the increased accumulation of toxic elements present in the leaves of plants^73^.

Low photosynthesis causes lower amounts of assimilates, thus leading to limited biomass^74^. In this study, a significant decrease in free AAs, especially Glu, Pro and Orn, was observed (Table 3). Proline, which is synthesized from Glu or Orn^1,75^, is an essential AA for the synthesis of proteins rich in Pro or Hyp. These proteins are contained in the cell wall and are associated with cell growth, cell wall structure functionality and pollen fertility^75,76^. In contrast, the importance of Glu in plants results from the biosynthesis of chlorophyll and glutathione, which are subsequently used for the biosynthesis of phytochelatins. These thiol nonprotein metabolites subsequently detoxify the risk elements. Glutathione is an essential metabolite of the plant antioxidant system, which includes the ascorbate–glutathione cycle^77^. Glucose produces ascorbic acid, which is an irreplaceable component of the antioxidant system. Through glucose, the antioxidant system of plants is closely linked through the Calvin cycle to photosynthesis^63^. Under oxidative stress caused by toxic elements, a lack of ascorbic acid and Glu cause the antioxidant plant system to collapse. Glutamic acid, through the biosynthesis of glutathione and phytochelatins, is irreplaceable for the functioning of the plant's defence system and the detoxification of the toxic effects of risk elements^78^. Therefore, in lettuce leaves, the decrease in free AA was related to the defence response of the plants to oxidative stress, as evidenced by the increase in MDA (Table 3, Fig. 2a).

## Conclusions

The results of our study showed that contamination by very low doses of soil dust PM had no significant or direct effect on plant fitness. However, the lettuce leaf condition was affected by risk elements in soil dust PM. The degree of damage caused by oxidative stress was demonstrated by the increased level of MDA. The effect of oxidative stress caused by the increased content of toxic elements in lettuce leaves did not have a significant effect on the fitness of the plants because the decreases in DW, most photosynthetic parameters and pigments were not significant. The reduction of transport AAs, especially Glu, is related to the protection of plants against the increased oxidative stress caused by the accumulation of risk elements. The reduction in phenolic substances, especially the increase in the 5mC percentage, showed that the plants overcome oxidative stress caused after further accumulation of risk elements in soil dust PM. Therefore, this manuscript draws attention to the danger of soil dust PM containing risk elements through induced epigenetic changes in the tested plants. We propose the use of epigenetic changes as biomarkers of the possible change in activation of a stressed plant’s metabolism caused by environmental pollution.

## Data Availability

The data used to support the findings of this study are included within the article. The datasets generated and/or analysed during the current study are not publicly available due apprehension of unauthorized use of data and plagiarism but are available from the corresponding author on reasonable request.
